# Consistency of the Disposition Index in the Face of Diet Induced Insulin Resistance: Potential Role of FFA

**DOI:** 10.1371/journal.pone.0018134

**Published:** 2011-03-30

**Authors:** Darko Stefanovski, Joyce M. Richey, Orison Woolcott, Maya Lottati, Dan Zheng, Lisa N. Harrison, Viorica Ionut, Stella P. Kim, Isabel Hsu, Richard N. Bergman

**Affiliations:** Department of Physiology and Biophysics, Keck School of Medicine, University of Southern California, Los Angeles, California, United States of America; University of Bremen, Germany

## Abstract

**Objective:**

Insulin resistance induces hyperinsulinemic compensation, which in turn maintains almost a constant disposition index. However, the signal that gives rise to the hyperinsulinemic compensation for insulin resistance remains unknown.

**Methods:**

In a dog model of obesity we examined the possibility that potential early-week changes in plasma FFA, glucose, or both could be part of a cascade of signals that lead to compensatory hyperinsulinemia induced by insulin resistance.

**Results:**

Hypercaloric high fat feeding in dogs resulted in modest weight gain, and an increase in adipose tissue with no change in the non-adipose tissue size. To compensate for the drop in insulin sensitivity, there was a significant rise in plasma insulin, which can be attributed in part to a decrease in the metabolic clearance rate of insulin and increased insulin secretion. In this study we observed complete compensation for high fat diet induced insulin resistance as measured by the disposition index. The compensatory hyperinsulinemia was coupled with significant changes in plasma FFAs and no change in plasma glucose.

**Conclusions:**

We postulate that early in the development of diet induced insulin resistance, a change in plasma FFAs may directly, through signaling at the level of β-cell, or indirectly, by decreasing hepatic insulin clearance, result in the observed hyperinsulinemic compensation.

## Introduction

In 1981 our group pioneered the concept that there is a specific relationship between insulin secretion and insulin action that can be described by a rectangular hyperbola [1]. According to the “hyperbolic law” [2], the product of insulin secretion and insulin sensitivity approximates a constant, termed **disposition index (DI)**. The hyperbolic relationship suggests that an environmental change in insulin sensitivity (i.e. obesity, exercise) will be well compensated by a change in insulin secretion. For example, the onset of obesity has been shown to be associated with insulin resistance. [3], [4]. Furthermore, it was shown that obesity is correlated with fasting hyperinsulinemia [5].

Even with the abundance of evidence documenting hyperinsulinemic compensation for insulin resistance in rodent, dog and human studies, the precise mechanism by which a normal organism detects insulin resistance and compensates with hyperinsulinemia remains unknown. Terauchi et al. and colleagues showed that hyperinsulinemic compensation was absent in glucose kinase (Gck) deficient mice with FFA plasma levels matched to high fat fed controls [6]. Given that glucose kinase is an important enzyme in the pancreas glucose sensing pathway, Weir and colleagues concluded that glucose is the dominant signal in hyperinsulinemic compensation for insulin resistance [7]. Despite the plethora of studies showing the presence of normoglycemia in the hyperinsulinemic state [8], [9], Weir contends that the change in insulin sensitivity and the accompanying change in insulin secretion can be explained by glucose signaling [7]. Nevertheless, Steil and colleagues showed that chronic oversupply of both glucose and FFAs in the rodent model leads to a robust increase in beta-cell mass accompanied by hyperinsulinemia [10]. Interestingly, Carpentier and colleagues have shown that prolonged elevation of plasma FFAs leads to impaired secretion of insulin in nondiabetic obese humans [11]. Thus, these data provide evidence that perhaps the early change in plasma glucose or FFA produces chronic changes in insulin secretion. Therefore, it can be argued that due to the importance of nutrient homeostasis, an early transient change in plasma glucose or FFA that is quickly normalized might induce a robust and prolonged insulin secretion response.

Our group has reported that after six weeks of hypercaloric high-fat diet, dogs develop insulin resistance that is countered with hyperinsulinemic compensation [12]. After six weeks of fat feeding, there was no measurable change in fasting glucose, or fasting FFAs. However, there was a significant rise in *nocturnal* FFA plasma levels and no change in *nocturnal* plasma glucose after six weeks of high-fat feeding in the face of hepatic insulin resistance. Due to the evident occurrence of hyperinsulinemic compensation and the significant rise in nocturnal plasma FFAs, the possibility arose that the change in nocturnal FFAs could be a signal for hyperinsulinemic compensation. Thus, our study was in accordance with previous findings by Morgan and colleagues that showed the diurnal pattern of plasma FFAs [13]. However, our previous study did not exclude the possibility that an earlier-week rise in fasting glucose, FFAs or both could potentially augment insulin secretion. Thus, preliminary data suggests that glucose might not be the signal for hyperinsulinemic compensation. In contrast, nocturnal plasma FFAs emerge as a possible sole signal for high fat diet-induced hyperinsulinemic compensation.

We felt that there was a need to examine longitudinally the role of early-week changes in glucose, FFA or both as potential signals for compensatory hyperinsulinemia associated with high-fat diet induced insulin resistance. We hypothesized that the onset of insulin resistance would be closely followed by a change in plasma FFAs, and may result in changes of plasma glucose. The change in plasma FFAs alone or in conjunction with the change in plasma glucose could signal an increase in insulin secretion and consequent hyperinsulinemic compensation. Thus, we expected DI to remain constant throughout the six weeks study period.

## Methods

### Ethics Statement

All experimental protocols were approved by the USC Institutional Animal Care and Use Committee.

### Experimental Design

Prior to initiation of the hypercaloric high fat diet (baseline studies), every animal was subjected to a FSIGT protocol to assess SI, AIRg, Sg, and DI using MINMOD, the computer program for estimating the parameters of Bergman’s Minimal Model [14]. The baseline FSIGTs were followed by evening and nighttime sampling of plasma glucose and FFAs during two time periods (6–8 PM, 2–4 AM) during the course of one day, a week before initiating hypercaloric high fat feeding. Also, we conducted baseline MRIs to assess body composition. Once the baseline studies were completed the hypercaloric high fat diet was initiated for a period of six weeks. For each animal we performed bi-weekly FSIGTs throughout the fat feeding regimen for a total of six weeks. At week six, we once again measured plasma glucose and FFA during the two time periods mentioned above. In addition, a second set of MRI’s was conducted at the end of the study to assess changes in body composition.

### Animals

All experiments were conducted on male mongrel dogs in the conscious relaxed state. Animals were housed under controlled kennel conditions (12-h light/dark cycle) in the University of Southern California (USC) Medical School Vivarium. Animals were accepted into the study following physical examination and a comprehensive blood panel. Dogs were used for experiments only if judged to be in good health as determined by visual observation, body temperature, and hematocrit.

### Diet

Dogs were fed a weight-maintaining standard diet of one can of Hill’s Prescription Diet (415 g, 10% carbohydrate, 9% protein, 8% fat, 0.3% fiber, and 73% moisture [Hill’s Pet Nutrition, Topeka, KS]) and 825 g dry chow (40% carbohydrate, 26% protein, 14% fat, and 3% fiber [mixture of Laboratory High Density Canine Diet and Prolab Canine 2000, Richmond, IN]) for a period of 2–3 weeks prior to conducting any experiments to monitor weight stabilization. This standard diet consisted of 3880 kcal/day: 38% from carbohydrates, 26% from protein and 36% from fat. Following weight stabilization, dogs were switched to hypercaloric, high fat diet for a period of 6 weeks in which the standard diet was supplemented with 6 g per kg of pre-diet body weight cooked bacon grease supplied by the Keck School of Medicine cafeteria. This hypercaloric, high fat diet consisted of 5392 kcal/day: 27% carbohydrates, 19% protein and 53% fat. All animals were presented with the food at approximately 9 AM each morning and the food was withdrawn at 12 PM. On days when experiments were conducted, the food was presented to the dogs upon their return to the vivarium and it was withdrawn after 3 hours.

### FSIGT

The FSIGTs were performed as previously described [15]. Glucose and insulin doses were determined based on the animal’s body weight the day before the experiment. Animals were familiarized with the Pavlov sling at least one week before the first FSIGT. At approximately 7 AM on the day of the FSIGT, animals were brought into the laboratory and placed in the Pavlov sling. A 19-gauge angiocatheter was placed in a peripheral vein and secured. Approximately 20 min later the first fasting sample was taken. After three fasting samples (−20, −10, and −1 min), 0.3 g/kg of glucose (50% dextrose, 454 mg/ml) was injected into the peripheral vein (t = 0). Subsequently, insulin was injected at t = 20 min (0.03 U/kg porcine insulin; Eli Lilly and Company, Indianapolis, IN, USA). Additional blood samples at t = 2, 3, 4, 5, 6, 8, 10, 12, 14, 16, 19, 22, 23, 24, 25, 30, 40, 50, 60, 70, 80, 90, 100, 110, 120, 140, 160, and 180 min were taken for assay of glucose, FFA, C-peptide and insulin.

### Plasma Glucose and FFAs

Fasting values were defined as the average of the three samples (−20, −10, and −1 min) before the glucose bolus. In addition to the fasting samples we obtained 10 additional samples of plasma glucose and FFAs measured within two time intervals (6–8 PM, 2–4 AM).

### Blood Sampling

Samples for determination of glucose and insulin samples were placed into 1.7 ml chilled tubes coated with lithium fluoride and heparin containing 50 µl EDTA. FFA samples were taken in 1.7 ml tubes with 25 µl EDTA and 50 µl paraoxon to inhibit lipase activity. C-peptide was collected into tubes containing 25 µl EDTA, 50 µl Trasylol. All samples were immediately centrifuged, and plasma separated. All samples were stored at −80°C until analysis.

### Assays

Glucose was measured in duplicate with a YSI 2300 autoanalyzer (Yellow Springs Instruments; Yellow Springs, OH, USA). Free fatty acids (NEFA C, Wako Pure Chemical Industries; Richmond, VA, USA) were measured using colorimetric methods in commercially available kits. Insulin was measured by an ELISA originally developed for human serum or plasma (Linco Research, St. Charles, MO, USA) and adapted for dog plasma in our laboratory. The method is based on the two murine monoclonal antibodies that bind to different epitopes of insulin, but do not bind to proinsulin. C-Peptides are measured using radioimmunoassay kits (Millipore Corporation, Billerica, MA).

### Magnetic Resonance Imaging (MRI)

Magnetic resonance imaging (MRI) scans were performed on the dogs as previously described [16]. Thirty 1-cm axial abdominal images (T1 slices; TR 500 TE:14) were obtained using a General Electric 1.5 Tesla Horizon (v5.7 software) magnet. Of the 30 images obtained, approximately 20 were used for analysis of total trunk body fat, depending on the relative torso length of the animal. Images were analyzed using Scion Image (Windows 2000 Version Beta 4.0.2; Scion Corporation, Frederick, MD, USA), which quantifies fat tissue (pixel value 121–254) and other tissue (20–120) in each slice. Fat volume was calculated by dividing the number of pixels counted as fat by the ratio of the total number of pixels (256×256) and known area (34.9 cm × 34.9 cm) for a 1-cm image. Total trunk fat and tissue was estimated as the integrated fat or tissue across all 20 slices. Percent fat was calculated as the total trunk fat divided by the total trunk tissue. Omental fat is defined as fat within the peritoneal cavity in an 11-cm region of the thorax, using the slice at the level where the left renal artery branches from the abdominal aorta as a midpoint landmark. Percent omental fat was calculated as the omental fat divided by the total tissue area in these same slices.

### Calculations

#### Minimal model parameters

Insulin Sensitivity (SI) and glucose effectiveness (Sg) was calculated by fitting the glucose profiles from the FSIGTs using Minmod Millennium (Version 6.02, 2004), MINMOD Inc., Los Angeles, CA. AIRg was calculated as the AUC of the insulin concentration above the average of the basal value, from 0 to 10 min after the glucose injection. DI, which represents a measure of insulin responsiveness corrected for changes in SI, was calculated as the product of the average SI and AIRg from each experiment.

#### Glucose Tolerance (Kg)

Glucose tolerance was assessed by the Kg parameter, which is calculated as the slope of the natural log of the glucose concentration vs. time from 8 to 19 min after the glucose bolus.

#### Metabolic Clearance Rate of Insulin (MCR)

The MCR of insulin (ml/kg/min) was calculated as the ratio of the amount of insulin (pmol/l) to the area under the fitted decay curve of insulin profile from t = 20 minutes (time of insulin bolus) to 60 minutes [17]. The fit of the model required to estimate the AUC and the calculation of MCR was obtained using WinSAAM, Mathematical Modeling Program, Kennett Square, PA.

### Statistical Analysis

All experimental data are expressed as means ± SE. General estimation equation with interaction expansion were used to compare all longitudinal data before and after fat feeding. Paired Student’s t tests was used to identify the changes in MRI determined total tissue, total fat tissue, visceral adipose tissue (VAT), subcutaneous adipose tissue (SAT), and non-fat tissue. Spearman rank correlation coefficient was used to calculate the correlations between two indexes. Most of statistical calculations were conducted using STATA, statistical package program, Stata Corp., State College, TX. T-tests was performed using Excel 2003, Microsoft, Redmond, WA.

## Results

### Body composition

**Figure 1:**
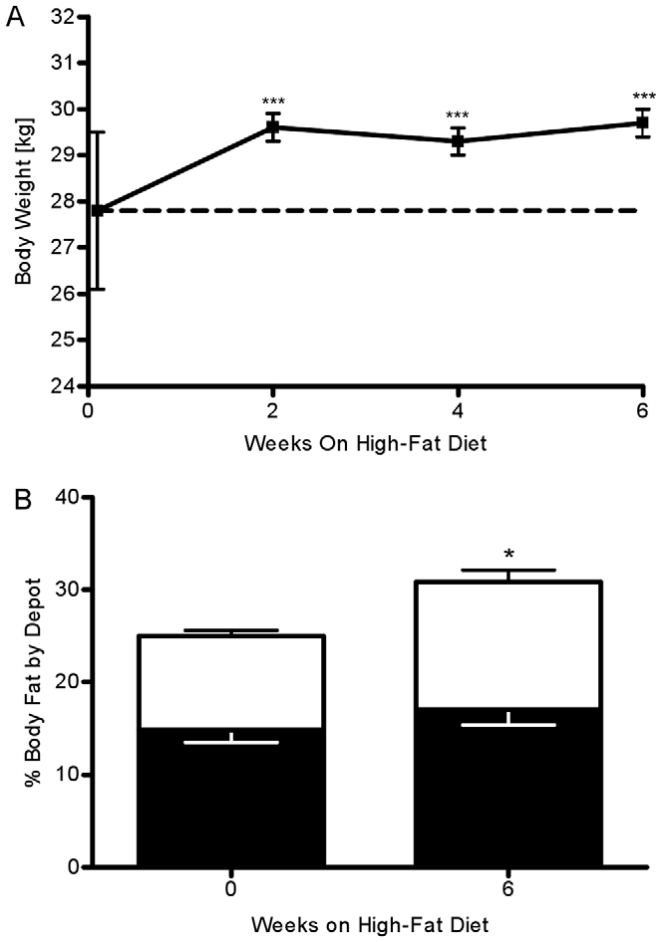
Weekly changes in body weight and body composition. (A) Body weight increased by week 2 and remained elevated throughout the six-week hypercaloric high-fat feeding period. (B) Visceral (white bar) and subcutaneous (black bar) adipose tissue calculated as area percent of total tissue. *P<.05 vs. week0; **P<.01 vs. week 0; ***P<.001 vs. week 0.

Mean body weight increased by 1.83±0.25 kg (P<0.001) at week 2 from 28.43±1.62 kg and remained elevated until the end of the study (Week 6: 1.89±0.25 kg, P<0.001, Fig. 1A). Weight gain was coupled with a 23.5±2.5 % (P = 0.02) increase in total trunk body fat tissue volume. This increase in body fat tissue was associated with both an increase in both visceral adipose tissue volume (VAT, 14.4±1.4 %, P = 0.06, Fig. 1B) and subcutaneous adipose tissue volume (SAT, 36.7±1.3 %, P = 0.02). Additionally, this increase in volume of adipose tissue was associated with an increase in adipose fat mass (VAT,20.3±3.2 %, P = 0.03; SAT, 43.8±5.6 %, P<0.01) There was no change in the volume or mass of non-fat tissue (data not shown).

### Fasting glucose and FFAs

**Figure 2:**
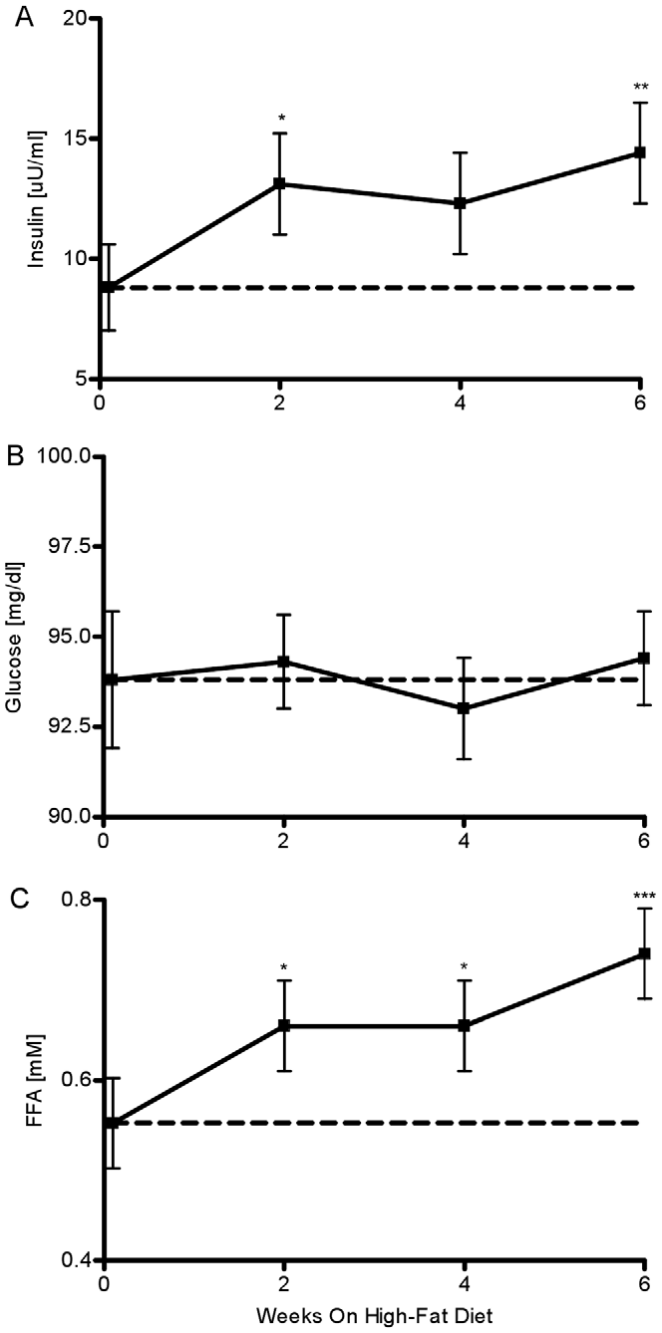
Observed fasting plasma insulin, glucose and FFAs. Fasting levels of (A) insulin, (B) glucose, and (C) FFA before and after fat feeding. *P<.05 vs. week0; **P<.01 vs. week 0; ***P<.001 vs. week 0.

Fasting plasma insulin increased by week 2 (week 0: 8.8±1.8 vs. week 2: 13.1±2 µU/ml, P = 0.038, Fig. 2A) and this increase was maintained throughout the six week study period (week 4: 12.3±2 µU/ml, P = 0.09; week 6: 14.4 µU/ml, P = 0.007). Hyperinsulinemia occurred despite absolutely no change in fasting plasma glucose (week 0: 93.8±1.9 vs. week 2: 94.3±1.3; P = 0.88, week 4: 93±1.4; P = 0.77, week 6: 94.4±1.3 mg/dl; P = 0.84, Fig. 2B). But, FFA were elevated by week 2 (week 0: 0.55±0.05 vs. week 2: 0.66±0.05 mM, P = 0.04, Fig. 2C). This increase was maintained through week 4 (P = 0.03). At week 6, fasting FFAs further increased by 35% (week 6: 0.74±0.05 mM, P<0.001).

### Evening and nocturnal glucose and FFAs

**Figure 3:**
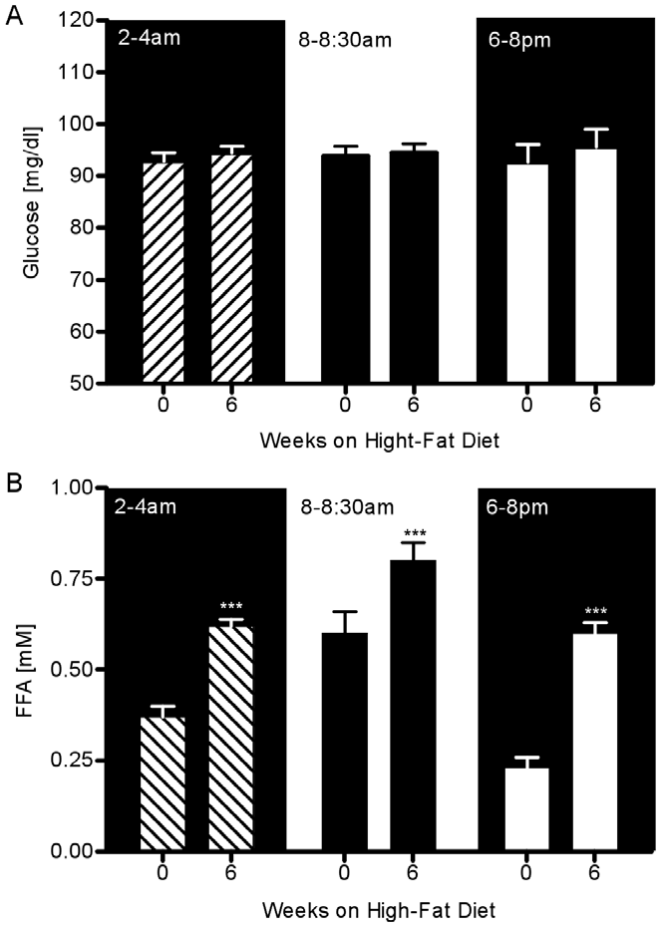
Glucose and FFA plasma levels at three time periods during 24-hr before and after fat feeding. (A) Plasma glucose and (B) plasma FFAs during 2–4 AM (hash bars), 8–8:30 AM (black bars), and 6–8 PM (white bars). *P<.05 vs. week0; **P<.01 vs. week 0; ***P<.001 vs. week 0.

At weeks 0 and 6, 10 additional samples of glucose and FFAs were obtained within two time intervals (6–8 PM, 2–4 AM). There was no change in the plasma glucose at the 6–8 pm interval before versus after six weeks of fat feeding (week 0: 92.7±3.4 vs. week 6: 95.5±3.5 mg/dl, P = NS, Fig. 3A). Also, there was no change in the plasma glucose in the time interval 2–4 AM (week 0: 92.8±1.7 vs. week 6: 94.4±1.3 mg/dl, P = NS). In contrast to glycemia, a 160% increase in evening FFAs was observed with fat feeding (week 0: 0.23±0.03 vs. week 6: 0.6±0.03 mM, P<0.001, Fig. 3B). A 68% increase in the nocturnal FFAs was observed with fat feeding (week 0: 0.37±0.03 vs. week 6: 0.62±0.03 mM, P<.001).

### Insulin Sensitivity (SI)

**Figure 4:**
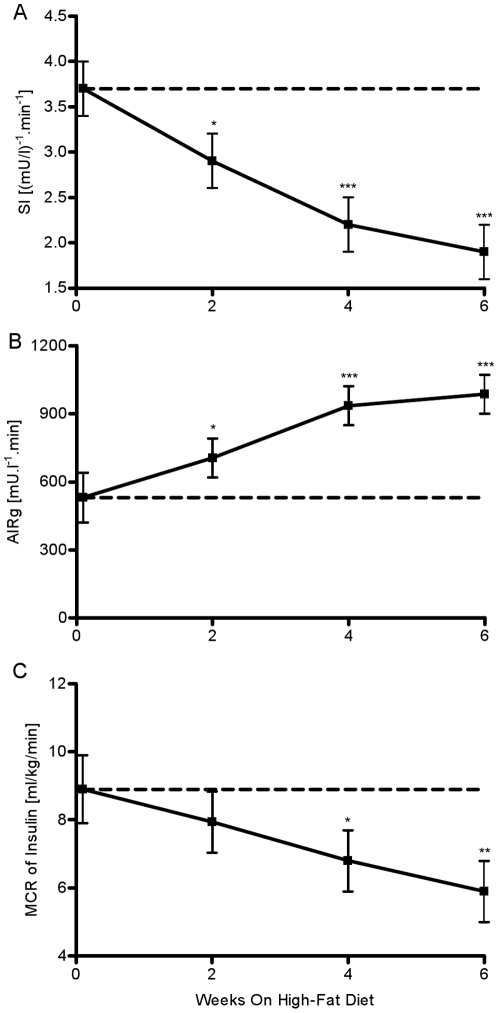
Weekly estimates of insulin sensitivity, insulin secretion and whole-body insulin clearance. (A) SI, (B) AIRg, and (C) MCR of insulin. *P<.05 vs. week 0; **P<.01 vs. week 0; ***P<.001 vs week 0.

The high fat diet caused SI to drop 22% by week 2 (week 0: 3.7±0.3 vs. week 2: 2.9±0.3 (mu/l)∧-1.min∧-1, P = 0.021, Fig. 4A). By week 4, SI was further reduced by 41% (P<0.001). At week 6, insulin sensitivity was reduced by 50% when compared to week 0 (week 6: 1.9±0.3 (mu/l)∧-1.min∧-1, P<0.001).

### MCR of Insulin

We observed a trend for a decrease in MCR of insulin by week 2 (week 0: 8.9±1 vs week 2: 7.94±0.9 ml/kg/min, P = NS, Fig. 4C). By week 4, the decrease became significant and more pronounced (week 4: 6.8±0.9 ml/kg/min, P = 0.016). Fat feeding caused a 33% decrease in MCR of insulin by week 6 (P = 0.001).

### Glucose Tolerance (Kg) and Disposition Index (DI)

**Figure 5:**
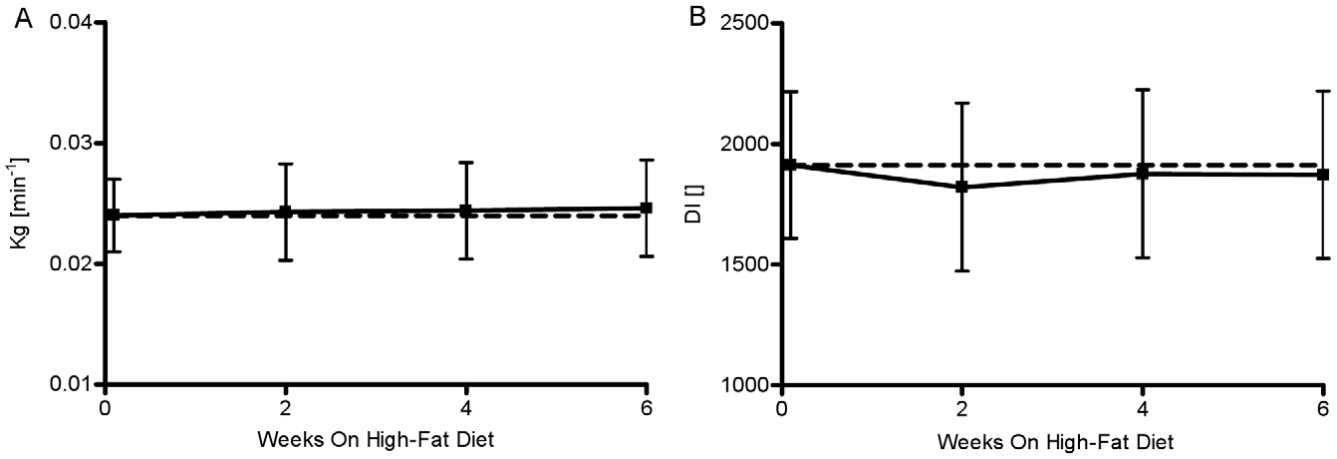
Weekly estimates of glucose tolerance and disposition index. (A) Glucose tolerance (Kg), (B) and disposition index (DI) over the 6 weeks of hypercaloric high-fat feeding period. *P<.05 vs. week 0; **P<.01 vs. week 0; ***P<.001 vs. week 0.

**Figure 6:**
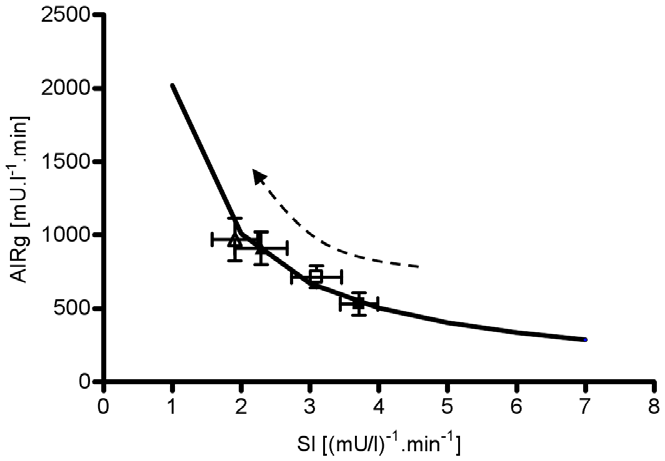
Consistency of the hyperbolic relationship (DI) between insulin sensitivity and insulin secretion. Solid black hyperbolic curve =  average DI value for week 0; black solid square  =  average DI for week 0; open square  =  average DI for week 2; black solid triangle  =  average DI for week 4; open triangle  =  average DI for week 6. The hyperbola represents the curve on which the animals would have to remain to maintain a constant DI (DI = AIRg.SI = constant) and it is calculated as extrapolation of the average DI for week 0 over a range of insulin sensitivities.

Glucose tolerance was not altered over the period of six weeks (P = NS, Fig. 5A). Additionally, DI remained constant over the study period (week 0: 1912.2±303, week 2: 1820.1±348, week 4: 1875.6±348, week 6: 1871.7±348, P = NS, Fig. 5B). The hyperbola on Figure 6 was generated from extrapolated values of insulin secretion (AIRg) based on observed value of DI for week 0 and varying SI in the range from1 to 7. All subsequent bi-weekly observations of SI and AIRg are represented by a graphical symbol with the respective SE (Fig. 6). Figure 6 shows that none of the bi-weekly observations significantly deviate from the hyperbola computed from the observed value of DI at week 0. Thus, this figure illustrates the complete hyperinsulinemic compensation for insulin resistance induced by fat feeding (Fig. 6).

### Correlations

**Figure 7:**
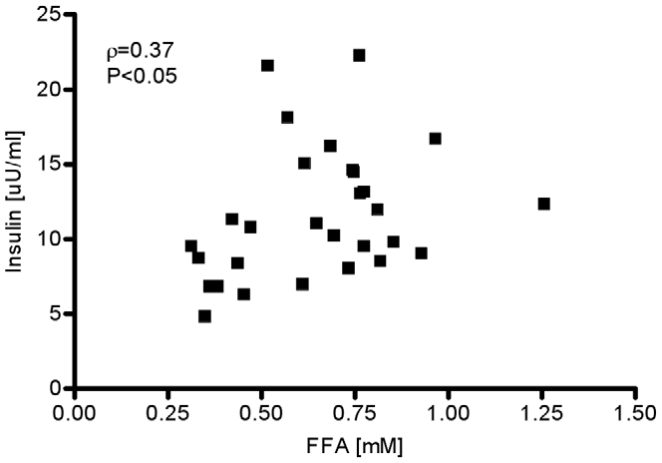
Spearman’s rank correlation between fasting insulin and fasting FFA.

Fasting FFAs were significantly correlated with change in fasting insulin (ρ = 0.37, P<0.05, Fig. 7).

## Discussion

The goal of this study was to examine the possibility that early-week changes in plasma glucose, FFAs, or both, are nutrient signals that lead to hyperinsulinemic compensation. To test our hypothesis, we utilized a model of canine obesity based on feeding dogs a hypercaloric, high-fat diet to induce the development of insulin resistance. The ensuing insulin resistance was countered with the onset of hyperinsulinemic compensation. The result of our observation was that the DI remained unchanged (Fig. 6). Thus, this study supports previous studies from our lab and others, demonstrating almost perfect maintenance of glucose tolerance in the face of insulin resistance, sustained by complete compensation for the ensuing resistance and constant Dl [18], [5].

Here we focused on examining the possible nutrient signals for the compensation. One potential candidate signal is the change in the plasma glucose level that develops as a consequence of reduced insulin sensitivity, which leads to diminished capability of cells to metabolize glucose, and a subsequent rise of glucose levels in the circulation. However, we found absolutely no change in morning fasting glucose over the period of six weeks (Fig. 2B) and no measurable changes in plasma glucose levels at two additional time periods (6–8 pm and 2–4 am) (Fig. 3A). This was not surprising finding since our previous studies have shown that dietary interventions in the canine model result in no changes in EGP and only mild peripheral insulin resistance, two factors that may lead to elevated plasma glucose [16], [12], [19]. Thus, the fact that basal EGP is unchanged with high-fat feeding combined with relatively unchanged peripheral disposal may explain the fact that fasting, 6–8 pm, and 2–4 am plasma glucose levels remained unchanged (Fig. 2B, Fig. 3A). These findings are in concordance with previous studies in which dog or human populations in the pre-diabetic state were able to maintain normoglycemia despite the onset of insulin resistance [5], [12], [8], [9].

Another possible signal for hyperinsulinemic compensation can be the change in plasma FFA levels. Our group and others have shown significant changes in the nocturnal FFA levels in the insulin resistant or diabetic state [20], [13], leading to the hypothesis that perhaps the change in plasma FFA levels, and not plasma glucose, may be the signal for hyperinsulinemic compensation [21], [22]. In this study we focused on the possibility that earlier-week changes in plasma FFAs may be the signal for hyperinsulinemic compensation. Our study confirmed that evening and nocturnal FFAs were significantly elevated by week 6 when compared to the observations at the same time interval at week 0. At week 6, evening FFAs showed a 160% increase (P<0.001), while nocturnal FFAs had a 68% increase (P<0.001) when compared to week 0. We did not conduct bi-weekly sampling of evening and nocturnal FFAs because the night time sampling protocol can be disruptive to the animal’s daily routine. Disrupting the rest/sleep pattern may lead to additional insulin resistance, which may be associated with other factors, such as sleep deprivation, and not necessarily caused by increased nutrient flow [23]. Since the rise in nocturnal FFAs is a slow process, we assumed that the subsequent increase in nocturnal FFAs would also be a gradual process. Therefore, we would be able to measure partial changes in plasma FFAs by collecting the fasting samples early in the morning. Our study identified an earlier-week rise in fasting FFAs as another potential signal. We observed a statistically significant rise in fasting FFAs at week 2 (20% increase, P = 0.04) that was sustained throughout the study period (Fig. 2C). At week 6, plasma FFAs were increased by 35% (P<0.001).

There may be three explanations for this rise in plasma FFA levels. First, since the diet was rich in fat (53% of the total calories were from fat), it is reasonable to postulate that the higher availability of FFAs may lead to the rise in plasma FFAs. Second, the hypercaloric, high-fat diet led to a 23% rise in total adipose tissue by week 6, which could lead to elevated FFA levels. It has been shown that the increased size of the adipose tissue depot leads to an elevated lipolysis rate, especially in the visceral adipose tissue (VAT) that drains directly into the portal vein [24], [25], [26], [27]; thus, a trend of increase in the VAT is another possible explanation for the rise in FFA levels. Third, it is possible that the increase in plasma FFA levels is due to increased sympathetic nervous system (SNS) activity. SNS activity is markedly increased in the insulin resistant state such as obesity and Type 2 diabetes [28].

Examination of the profiles of the weekly changes in fasting insulin and early-morning fasting FFAs in our study exhibited remarkable similarities (Fig. 2A, C). The bi-weekly average rate of change in fasting insulin was very similar to the rate of change in fasting FFA. In contrast, this pattern was missing when fasting glucose was considered (Fig. 2B). Statistical analysis of the data revealed a significant correlation between weekly changes in fasting insulin and changes in FFAs (Fig. 7B, ρ = 0.38, P<0.05).The previously noted strong visual similarities between weekly changes in insulin and early morning FFAs seem not to be confirmed with the moderately statistically significant correlation. One possibility for the lack of a much stronger correlation could be due to variability in both insulin and FFAs. It is well established that both insulin and FFAs are released in a pulsatile manner [29]. Therefore, the high variability may be a consequence of the fact that our measurements may reflect both peaks and troughs of insulin and FFA release. Another possibility is that the early morning plasma FFAs are not appropriate surrogates for nocturnal plasma FFA levels. From figure 3B it is evident that the increase of 65% in nocturnal plasma FFA level at week 6 is higher than the change of 35% in the early morning FFA levels. Thus, we believe that high variability in insulin and FFA, which is more than likely attributed to the pulsatile release of these substrates combined with the attenuated change in early morning plasma FFA levels, resulted in a moderate correlation. Hence, we maintain that the increase in nocturnal plasma FFAs observed during the development of obesity may represent a signal for hyperinsulinemic compensation.

Elevated plasma FFA levels have previously been shown to be related to insulin resistance [30]. It has also been shown that an increase in FFAs leads to alterations in insulin clearance [31]. Additionally, we have shown that the fat-fed canine model of obesity has a decreased first-pass hepatic extraction [32], which will lead to elevated plasma insulin. Consistent with this notion are the results from our study, which show that by week 4 of hypercaloric, high-fat feeding MCR of insulin was significantly reduced by 23% (P = 0.016). By week 6, MCR of insulin was reduced by 33% (P = 0.001). Therefore, it is possible that the observed early-week increase in nocturnal FFAs will lead to increased plasma insulin levels by altering the metabolism of insulin in the liver. Interestingly, many studies fail to identify the changes in insulin clearance as a physiologic adaptation that in combination with increased insulin secretion results in elevated plasma insulin levels. Another possibility is that the growth of the adipose visceral depot leads to an elevation in lipolysis and elevated FFAs levels in the portal vein. Increased influx of FFAs are well correlated with hepatic insulin resistance [33]. During a meal, the pancreas senses the rapid rise in plasma nutrient levels and releases a burst of insulin, which directly or indirectly result in complete suppression of EGP. The onset of obesity associated with the rise in plasma FFA levels and hepatic insulin resistance may prevent complete suppression from occurring[16]. Due to the continuous endogenous and exogenous supply of glucose, plasma glucose levels would remain elevated with the pancreas producing more insulin to renormalize glucose levels. Longitudinally, this anomaly might lead to an increase of beta cell mass and consequent hyperinsulinemia.

Previous studies suggest an interaction between glucose and free fatty acids with normal beta cell function, which raises the possibility that imbalances between the two fuels *in vivo* can have pathological consequences, such as impaired glucose-stimulated insulin secretion (GSIS)[34]. Nolan and colleagues hypothesized three potential mechanisms through which FFAs in conjunction with glucose may lead to amplification of GSIS [21]. Two of the pathways implicate beta cell intercellular metabolism of FFAs, whereas, the third is related to membrane free fatty acid receptor (FFAR) activation. Regardless, a change in plasma FFAs can be detected by the β-cell through the G-protein-coupled receptor GPR40/FFAR1 without any changes in plasma glucose levels and may lead to increased secretion of insulin [20]. Thus, in the absence of plasma glucose changes, which were observed in our study, FFAs can act as the signal leading to increased GSIS.

In summary, six weeks of a hypercaloric, high-fat diet induced severe insulin resistance in the canine model. Even in the face of extreme insulin resistance, DI remained unchanged due to complete hyperinsulinemic compensation. Here we offer additional evidence of the temporal sequence for hyperinsulinemic compensation. **Our data strongly suggests that the early evolution of obesity-induced elevation of nocturnal FFA may directly modulate GSIS in the pancreas and/or indirectly reduce hepatic insulin clearance, either mechanism serving as a potential hyperinsulinemic compensatory signal.**

To effectively prevent the onset of diabetes, it is pertinent to understand the early changes in the pre-diabetic state. Our results indicate that in the case of complete hyperinsulinemic compensation for insulin resistance there were no observable changes in ambient plasma glucose. Therefore, it is plausible that when glucose levels change, such as in impaired glucose tolerance (IGT), some of the compensatory mechanisms may have been irreversibly lost. Hence, screening early morning fasting FFA or insulin may be an effective strategy in early detection of subject with high risk for development of diabetes. However, due to the fact that currently there are no available standards for measuring both insulin and FFA we believe that our proposed screening strategy may be a viable option in the future.
